# 

**Published:** 1897-10

**Authors:** 


					﻿Vol. XVII.
OCTOBER, 1897.
NO. 16.'
THE
HOMEOPATHIC pHY^lClAjy,
A Monthly Journal of Homoeopathic Materia Medica
and Clinical Medicine.
“ He it a freeman whom truth makes free, and all are slaves beside."
"Seek the Truth: come whence it may, cost what it will.”
EDITED AND PUBLISHED BY
WALTER M. JAMES, M. D.
PHILADELPHIA:
No. 1231 LOCITST STREET.
lowdon:
ALFRED HEATH & CO., Sole Agents fob Gbeat Britain,
114 Ebury Street, Eaton Square.
■Yearly Subscription, $2.SO, invariably in advance. Single numbers, 2S els.
Entered at the Philadelphia Post-Office aa Second-Cl aai Mail Matter.
				

